
JOURNAL INFORMATION
==============================
NLM Title Abbreviation: ScientificWorldJournal
Journal ID: ScientificWorldJournal
NLM Journal ID: 101131163
Journal ID: TSWJ

The Scientific World Journal

ISSN: 2356-6140
EISSN: 1537-744X
Publisher: Wiley

ARTICLE INFORMATION
==============================
PMCID: PMC5763706
PMID: 20305984
DOI: 10.1100/tsw.2010.53
Article ID: 323104
Article version: 1
Subjects: Editorial

In and Out — Biochemical and Biophysical Mechanisms of Viral Fusion and Assembly

Santos Nuno C. *
Instituto de Medicina Molecular, Faculdade de Medicina da Universidade de Lisboa, Lisboa, Portugal
*Nuno C. Santos: nsantos@fm.ul.ptAcademic Editor: Claire Redhead

Publication date: 2010
Electronic publication date: 2010 Mar 16
Volume: 10
First page: 419
Last page: 421
Received 2010 Mar 3; Accepted 2010 Mar 4
Copyright: Copyright © 2010 Nuno C. Santos.
Copyright year: 2010
License: This is an open access article distributed under the Creative Commons Attribution License, which permits unrestricted use, distribution, and reproduction in any medium, provided the original work is properly cited.
License URL: https://creativecommons.org/licenses/by/3.0/

==============================
Editorial
Special Issue: Biochemical and Biophysical Mechanisms of Viral Fusion and Assembly
*TheScientificWorldJOURNAL* (2010) 10, 419–421
ISSN 1537-744X; DOI 10.1100/tsw.2010.53

TheScientificWorldJOURNAL
www.thescientificworld.com

### In and Out – Biochemical and Biophysical Mechanisms of Viral Fusion and Assembly

Nuno C. Santos

*Instituto de Medicina Molecular, Faculdade de Medicina da Universidade de Lisboa, Lisboa, Portugal*

**E-mail**: nsantos@fm.ul.pt

*Received March 3, 2010; Accepted March 4, 2010; Published March 16, 2010*

---

Viruses are unable to replicate by themselves, making it mandatory to exploit the replication machinery of a target cell. To accomplish this, it is first necessary for the virus to recognize an "infectable" cell and overcome its first defense barrier – the cell membrane. Different viruses have different strategies to accomplish this purpose, despite several common aspects. After viral entry and replication of its genetic information (either as DNA or RNA), the viral assembly process culminates with a new stage of interaction with the cell membrane in order for the new virions or viruses to be released to the extracellular media. The biochemical and biophysical aspects of these stages are the main focus of this special issue of *TheScientificWorldJOURNAL*.

In this special issue dedicated to the *Biochemical and Biophysical Mechanisms of Viral Fusion and Assembly*, the review article by Wessels and Weninger[1] is centered on the entry of enveloped viruses (such as influenza virus or HIV) onto a target cell, describing the two viral players on this process: viral membrane proteins and lipids. Large conformational changes in the proteins embedded in the viral membrane play a fundamental role in the membrane fusion process. The authors describe the different viral entry mechanisms, showing that, despite the tremendously wide variety of enveloped viruses, they all contain membrane fusion protein machinery with a remarkably conserved mechanism of action. The functional commonality among the three classes of viral fusion proteins is surprising considering the diversity of structures and the varied fusion triggers. In this article, the focus on the role of lipids in membrane fusion describes the lipid biochemistry and biophysics aspects necessary for the fusion between viral and target cell membranes to occur, keeping in mind some of the experimental techniques used to identify those requirements. In that context, strong emphasis is given to X-ray crystallography, molecular dynamics, conductivity, and fluorescence spectroscopy measurements.

While Wessels and Weninger dealt with the entry of enveloped virus onto a target cell[1], Ruiz et al.[2] focused on the cellular entry of rotavirus (a nonenveloped virus), following a complex multistep process in which different domains of the rotavirus surface proteins interact with different cell surface molecules, which act as putative receptors on the plasma membrane. Beside the different aspects of rotavirus entry (viral structure, cell receptors, penetration, permeabilization, membrane interaction, and endocytosis), the article integrates the knowledge of other steps of the viral life cycle; namely, replication and assembly. The relevance of calcium ion and its homeostasis for viral infection and pathogenesis is worthy of notice. Its description largely benefits the expertise of the authors on this specific subject[3,4,5].

The article by Huerta et al.[6] is focused on the process of membrane fusion between HIV-infected cells and noninfected target cells. It is promoted by the HIV envelope glycoprotein complex formed by gp120 and gp41, and can lead to the formation of multinucleated cells, called syncytia. Even in the asymptomatic stage of HIV infection, multinucleated cells have been observed in different organs, indicating that fused cells may be generated and remain viable in the tissues of patients. The authors

©2010 with author.
Published by TheScientificWorld; www.thescientificworld.com developed a method for the direct quantification of fusion events by flow cytometry, involving the staining of fusion partners with lipophilic probes and the use of fluorescence resonance energy transfer (FRET) to distinguish between fused and aggregated cells[7,8]. Its application indicates that the time of reaction and initial proportion of fusion partners determine the number, relative size, and average cellular composition of syncytia. This quantitative approach, fully explained in the present article, has also shown that the heterogeneity of syncytia generated by envelope proteins–mediated cell-cell fusion may result in a variety of possible outcomes that, in turn, may influence the biological properties of the syncytia and surrounding cells, as well as viral replication.

While the previously mentioned articles in this special issue deal mainly with the entry of the viral content onto a target cell[1,2,6], the article by José L. Neira[9] is focused on one of the key players of the HIV assembly process – the capsid protein. The ordered oligomerization of several hundred copies of this protein constitutes the scaffold for the new virion formation, enclosing the viral RNA and other proteins. The article fully reviews the role of the C-terminal domain of the capsid protein, CAC, as the driving force for viral assembly, its biophysical studies, its interaction with other proteins or lipids, and the promising development of drugs able to hamper capsid protein assembly, dimerization, or proper folding. Taken together, the results described suggest that the multiple roles of the HIV-capsid protein during viral assembly may be mediated by its rather unique ability to adopt different conformations, while preserving a similar supramolecular organization in response to different molecular environments and/or the interaction with other biomolecules.

Finally, the article by Santos et al.[10] presents a combined effort from clinicians and researchers to elucidate the changes in several biophysical and biochemical parameters of the blood cells of HIV-infected patients. In a previous paper[11], the authors showed that lymphocytes and erythrocytes of HIV-1–infected patients, before antiretroviral therapy, presented significant changes in intracellular calcium concentration ($[Ca^{2+}]_{int}$) and membrane fluidity. In the present study, the same parameters were evaluated after efficient response to highly active antiretroviral therapy (HAART). When compared with the control group, both untreated and treated patients presented increased lymphocyte $[Ca^{2+}]_{int}$ and decreased lymphocyte membrane fluidity, without significant differences between the two groups of patients. On the contrary, the therapy reversed the membrane fluidity variations observed on erythrocytes. The decreased erythrocyte $[Ca^{2+}]_{int}$ of untreated patients was not reversed by HAART. As shown, AIDS patients present changes in lymphocyte (mostly noninfected) and erythrocyte properties, partially reversed by HAART, consistent with a process of facilitated propagation of the infection to new cells (viral fusion), stimulation of virion assembly, and maintenance of a reservoir of erythrocyte-bound infectious virus. These observations can be related with the action of the HIV Nef protein on the cell's proteins and lipid composition, as well as with the recently observed cell infection by HIV-1 via endocytosis.

This last study was conducted primarily on cells that were not infected by HIV – erythrocytes and a great majority of the total lymphocyte population of HIV-infected patients. This is probably one of its main goals and innovations, as well as one of the most relevant aspects of its results. Usually, most of the studies focused exclusively on HIV-infected cells. This makes the average lymphocyte population of HIV-infected patients and, specially, their erythrocytes quite overlooked systems. Most of the lymphocytes of a HIV-infected patient and all the erythrocytes are not infected by the virus, but, as these are the majority of the cells in circulation in HIV-infected patients and considering also the multitude of effects of the infection in organs and tissues in which cells are not infected, those cells should not be ignored. Furthermore, it is important to bear in mind that HIV can be found not only in infected cells and free in the blood plasma, but also bound to other cells, including "noninfectable" lymphocytes[12,13] and erythrocytes[14,15], eventually constituting a reservoir of active virus in circulation.

### REFERENCES

1. Wessels, L. and Weninger, K. (2008) Physical aspects of viral membrane fusion. *TheScientificWorldJOURNAL* **9**, 764–780.

2. Ruiz, M.C., Leon, T., Díaz, Y., and Michelangeli, F. (2009) Molecular biology of rotavirus entry and replication. *TheScientificWorldJOURNAL* **9**, 1476–1497.
3. Michelangeli, F., Ruiz, M.C., del Castillo, J.R., Ludert, J.E., and Liprandi, F. (1991) Effect of rotavirus infection on intracellular calcium homeostasis in cultured cells. *Virology* **181**, 520–527.
4. Michelangeli, F., Liprandi, F., Chemello, M.E., Ciarlet, M., and Ruiz, M.C. (1995) Selective depletion of stored calcium by thapsigargin blocks rotavirus maturation but not the cytopathic effect. *J. Virol*. **69**, 3838–3847.
5. Ruiz, M.C., Cohen, J., and Michelangeli, F. (2000) Role of $Ca^{2+}$ in the replication and pathogenesis of rotavirus and other viral infections. *Cell Calcium* **28**, 137–149.
6. Huerta, L., López-Balderas, N., Rivera-Toledo, E., Sandoval, G., Gómez-Icazbalceta, G., Villarreal, C., Lamoyi, E., and Larralde, C. (2009) HIV-envelope-dependent cell-cell fusion: quantitative studies. *TheScientificWorldJOURNAL* **9**, 746–763.
7. Huerta, L., Lopez-Balderas, N., Larralde, C., and Lamoyi, E. (2006) Discriminating in vitro cell fusion from cell aggregation by flow cytometry combined with fluorescence resonance energy transfer. *J. Virol. Methods* **138**, 17–23.
8. Lopez-Balderas, N., Huerta, L., Villarreal, C., Rivera-Toledo, E., Sandoval, G., Larralde, C., and Lamoyi, E. (2007) In vitro cell fusion between $CD4^+$ and HIV-1 $Env^+$ T cells generates a diversity of syncytia varying in total number, size and cellular content. *Virus Res*. **123**, 138–146.
9. Neira, J.L. (2008) Biophysical and structural studies on the capsid protein of the Human Immunodeficiency Virus type 1: a new drug target? *TheScientificWorldJOURNAL* **9**, 404–419.
10. Santos, N.C., Martins e Silva, J., Freitas, T., Doroana, M., Duarte, N., Tavares, L., Antunes, F., and Saldanha, C. (2010) Blood cell membrane fluidity and intracellular $Ca^{2+}$ changes in antiretroviral-naïve and -treated HIV-1-infected patients. *TheScientificWorldJOURNAL* **10**, 350–355.
11. Martins-Silva, J., Santos, N.C., Doroana, M., Duarte, N., Tavares, L., Antunes, F., and Saldanha, C. (2006) Changes in blood cell membrane properties in HIV type-1-infected patients. *AIDS Res. Hum. Retroviruses* **22**, 849–853.
12. Olinger, G.G., Saifuddin, M., and Spear, G.T. (2000) CD4-negative cells bind human immunodeficiency virus type 1 and efficiently transfer virus to T cells. *J. Virol*. **74**, 8550–8557.
13. Moir, S., Malaspina, A., Li, Y., Chun, T.W., Lowe, T., Adelsberger, J., Baseler, M., Ehler, L.A., Liu, S., Davey, R.T., Jr., Mican, J.A., and Fauci, A.S. (2000) B cells of HIV-1-infected patients bind virions through CD21-complement interactions and transmit infectious virus to activated T cells. *J. Exp. Med*. **192**, 637–646.
14. Hess, C., Klimkait, T., Schlapbach, L., Del Zenero, V., Sadallah, S., Horakova, E., Balestra, G., Werder, V., Schaefer, C., Battegay, M., and Schifferli, J.A. (2002) Association of a pool of HIV-1 with erythrocytes in vivo: a cohort study. *Lancet* **359**, 2230–2234.
15. Horakova, E., Gasser, O., Sadallah, S., Inal, J.M., Bourgeois, G., Ziekau, I., Klimkait, T., and Schifferli, J.A. (2004) Complement mediates the binding of HIV to erythrocytes. *J. Immunol.* **173**, 4236–4241.

**This article should be cited as follows**:

Santos, N.C. (2010) In and out – biochemical and biophysical mechanisms of viral fusion and assembly. *TheScientificWorldJOURNAL* **10,** 419–421. DOI 10.1100/tsw.2010.53.
